# Genome-Wide Studies in Ischaemic Stroke: Are Genetics Only Useful for Finding Genes?

**DOI:** 10.3390/ijms23126840

**Published:** 2022-06-20

**Authors:** Cristina Gallego-Fabrega, Elena Muiño, Jara Cárcel-Márquez, Laia Llucià-Carol, Miquel Lledós, Jesús M. Martín-Campos, Natalia Cullell, Israel Fernández-Cadenas

**Affiliations:** 1 Stroke Pharmacogenomics and Genetics Group, Institut de Recerca de l’Hospital de la Santa Creu i Sant Pau, 08041 Barcelona, Spain; cristina.gallego.fabrega@gmail.com (C.G.-F.); elena.muinho@gmail.com (E.M.); jara.carcel@gmail.com (J.C.-M.); laialluciacarol@gmail.com (L.L.-C.); miquel.lledos@gmail.com (M.L.); jmartinca@santpau.cat (J.M.M.-C.); natalia.cullell@gmail.com (N.C.); 2 Institute for Biomedical Research of Barcelona (IIBB), National Spanish Research Council (CSIC), 08036 Barcelona, Spain; 3 Departament de Genètica i de Microbiologia, Universitat Autònoma de Barcelona, 08193 Bellaterra, Spain; 4 Stroke Pharmacogenomics and Genetics Group, Fundació MútuaTerrassa per la Docència i la Recerca, 08221 Terrassa, Spain

**Keywords:** stroke, GWAS, epigenetics, microbiome

## Abstract

Ischaemic stroke is a complex disease with some degree of heritability. This means that heritability factors, such as genetics, could be risk factors for ischaemic stroke. The era of genome-wide studies has revealed some of these heritable risk factors, although the data generated by these studies may also be useful in other disciplines. Analysis of these data can be used to understand the biological mechanisms associated with stroke risk and stroke outcome, to determine the causality between stroke and other diseases without the need for expensive clinical trials, or to find potential drug targets with higher success rates than other strategies. In this review we will discuss several of the most relevant studies regarding the genetics of ischaemic stroke and the potential use of the data generated.

## 1. Introduction

Stroke is a multifactorial disease, with diet, lifestyle, and genetics, amongst others, affecting both stroke occurrence and stroke outcome. It is estimated that one in every six people will suffer a stroke at some stage, thus making this the second or third most common cause of death worldwide. Moreover, stroke sequelae are associated with high rates of disability, and the corresponding increased impact on health care and economics due to population aging.

Over the last two decades we have observed a revolution in the genetics field, with the change from candidate gene studies to genome-wide association studies triggering a revolution in the research of complex diseases. The genome-wide era has resulted in the discovery of a multitude of genes and genetic variations associated with complex diseases such as stroke. Indeed, the results from genome-wide association studies (GWAS) have proved to be very robust and to present high reproducibility rates among different laboratories and genetic platforms. This robustness has been a key point in the use of genetic data to understand the biological mechanisms of complex diseases and to find potential treatments or drug targets, or to answer clinical questions without needing to perform a clinical trial.

In this review we will discuss the usefulness of the genetic data regarding stroke and the most interesting and recent advances in this field, including other techniques, such as epigenomics and microbiomics, that will play an important role in the near future.

## 2. Genetics of Ischaemic Stroke

**Figure 1:**
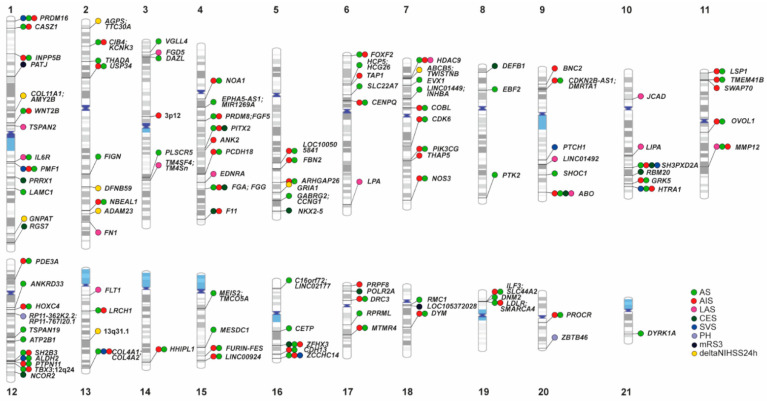
Genetic loci influencing stroke phenotypes. Ideogram of genomic regions influencing stroke phenotypes; colored circles represent genome-wide significant loci in published studies. Colors correspond to associated stroke phenotypes (green, AS: All Strokes; red, AIS: All Ischaemic Strokes; pink, LAS: Large-artery atherosclerosis Stroke; dark green, CES: Cardioembolic Strokes; blue, SVS: Small Vessel Stroke; purple, PH: Parenchymal Hematoma; black, mRS3: modified Rankin Scale three months after stroke; yellow, deltaNIHSS24 h: difference between NIH stroke scale (NIHSS) within six hours of stroke onset and NIHSS at 24 h. Prioritized genes in the original publications are displayed.

GWAS analysis in the field of ischaemic stroke (IS) risk has triggered a revolution in recent years. The first IS GWAS was published in 2007 and consisted of an analysis of about 500 IS cases and healthy controls [1]. Although this study did not find any significant signal, probably due to the small sample size, it was nevertheless the start of subsequent very successful GWAS analyses in this field. In 2016, the METASTROKE collaboration [2], which involved a GWAS meta-analysis comprising up to 10,307 cases and 19,326 controls, revealed four loci and subtypes associated with IS risk (*ABO*, *HDAC9*, *PITX2*, and *ZFHX3*). The *ABO* locus was found to be associated with IS and has previously been shown to be genome-wide associated with circulating levels of von Willebrand factor and factor VIII [3]. The *HDAC9* locus showed significance for large artery atherosclerosis stroke (LAS) and has also been linked to inflammation and atherosclerosis [4]. Finally, two loci associated with cardioembolic stroke (CES) risk, namely the priority genes *PITX2* and *ZFHX3*, are already known to be signals associated with atrial fibrillation risk [5], the most prevalent risk factor for CES. In the same year, and with a sample size of 16,851 cases and 32,473 non-stroke controls, the NINDS Stroke Genetics Network (SiGN) [6] found a new locus associated with LAS close to *TSPAN2* and confirmed the previous loci *ABO*, *HDAC9*, *PITX2*, and *ZFHX3*. In 2018, the MEGASTROKE Consortium published a GWAS meta-analysis with 67,162 cases and 454,450 controls [7]. In this analysis, 32 loci associated with stroke and its subtypes were found, confirming previous loci and revealing different novel loci (Figure 1). The loci found in the MEGASTROKE study showed previous associations with traits related to stroke risk, especially white matter hyperintensities in the brain, atrial fibrillation, intima-media thickness, blood pressure, coronary artery disease, lipid levels, and venous thromboembolism. Remarkably, 16 (11%) of the 149 genes located in the 32 stroke risk loci were found to be targets for currently approved drugs, especially antithrombotic therapies such as alteplase, tenecteplase, and cilostasol [7].

The same year, a joint GWAS meta-analysis between MEGASTROKE and The United Kingdom Biobank (UKB) (up to 72,147 cases and 823,869 controls) revealed three new loci associated with stroke (Figure 1) [8]. Of these, the *NOS3* gene reached significance only when considering the European population, whereas *COL4A1* and *DYRK1A* were identified in the multiethnic GWAS meta-analysis. These findings highlight the role of *COL4A1* in stroke. Indeed, this gene has previously been associated with small vessel disease [9]. In addition, the rs720470 variant identified is an eQTL (“expression quantitative trait loci”) of *DYRK1A*, which is involved in angiogenic responses in vascular endothelial cells [10]. With regard to *NOS3*, functional variants in this and other nitric oxide synthase (NOS)/nitric oxide (NO)) pathway genes have been associated with hypertension [11]. The authors of that study demonstrated how genetic variation in the NOS/NO pathway partly affects stroke risk as a result of variations in blood pressure by using the Mendelian Randomization approach [8].

A multiethnic GWAS meta-analysis by The Global Biobank Meta-Analysis Initiative (GBMI), including 19 biobanks and more than 2.1 million people, has been pre-published this year. In this study, 14 diseases, including stroke, were analyzed [12]. By combining both fixed-effects and inverse variance-based meta-analysis, the authors identified seven loci previously reported in stroke, along with nine new loci (Figure 1). For the *CENPQ* and *ALDH2* genes, a protein-coding variant was the most significant variant. Additionally, it was found that the *MEIS2*/*TMCO5A* gene locus was driven by variants that are more frequent in African ancestry. Furthermore, in a female-only meta-analysis, the previously reported *CETP* locus met the genome-wide significance threshold but did not reach significance in the combined sex meta-analysis [11]. Previous studies have shown that *CETP* transgenic expression increases plasma triglyceride levels in female and male mice by distinct mechanisms [13,14]. Finally, the authors of that study found pleiotropic associations for several of the identified genes with endocrine/metabolic, sense-organ, circulatory, and digestive-system phenotypes.

However, the largest step forward was taken by the GIGASTROKE project, which involved the largest multiethnic meta-analysis GWAS in stroke (110,182 stroke patients and 1,503,898 controls) [15], and in which the International Stroke Genetics Consortium (ISGC) collaborated [6]. The pre-printed results represent the most comprehensive description of stroke-risk genetic variants in Europeans, East Asians, South Asians, AfricanAmericans, and Hispanics to date. Thus, 89 loci, 61 of which are novel, were identified for stroke and stroke subtypes (Figure 1). The results suggest substantial shared susceptibility to stroke across populations. Cross-ancestry fine-mapping, in silico mutagenesis analysis with a machine learning approach, and transcriptome and proteome-wide association analyses was used to reveal putative causal genes and variants, such as *SH3PXD2A*, *FURIN*, and *NOS3*. Furthermore, the authors used a three-pronged approach to identify the putative drug targets for the prevention or treatment of stroke. Indeed, drugs targeting F11 and *PROC* are currently being explored in clinical trials [16]. Finally, polygenic risk scores (PGSs) integrating cross-ancestry and ancestry-specific stroke genetic risk variants with vascular risk factors were performed and enabled the prediction of ischaemic stroke in 52,600 participants with cardiometabolic disease. To sum up, the results of GIGASTROKE provide a better understanding of the pathogenesis of stroke and its subtypes, potential drug targets, and genetic risk prediction tools.

## 3. Genetics of Stroke Outcome

Stroke is the second leading cause of death worldwide [17,18]. Given the high global cost of stroke [17], even small improvements in recovery could have a major impact on health and cost reductions. The neurological changes during the first 24 h after the stroke (acute phase) largely determine the long-term evolution and disability [19]. As such, it is necessary to consider both the neurological evolution in the acute phase of stroke and the patient’s long-term functional status. Neurological evolution is measured using the National Institutes of Health Stroke Scale (NIHSS), and functional status is assessed three months after the stroke event, usually using the modified Rankin Scale (mRs) [19]. The outcome after an ischaemic stroke depends on multiple, complex, and interacting processes of brain tissue repair and injury, which are influenced by demographics, comorbidities, genetic factors, and external influences such as r-tPA (recombinant tissue-type plasminogen activator), and thrombectomy treatments [20]. The GWAS studies may allow us to uncover genes and pathways that shape the dynamic processes involved in stroke outcomes from early brain injury to long-term recovery. Despite the high number of genetic risk loci associated with stroke risk (>30 loci) [7], only a few have been associated with stroke outcome [21,22,23], thus suggesting that genetic factors related to stroke outcome are likely to be different from those influencing stroke risk. In addition, it has been observed that early neurological change after stroke measured using ΔNIHSS24 h (the difference between the NIHSS score at 24 h and the NIHSS score at baseline (less than 6 h from stroke onset)), has a different genetic architecture than the risk of IS [24].

The GENISIS (Genetics of Early Instability After Ischaemic Stroke) study performed a GWAS with 5876 individuals to examine neurological evolution in the acute phase of stroke using ΔNIHSS24 h. This study showed that common genetic variants accounted for 8.7% of the variance in ΔNIHSS24 h. The authors identified eight genome-wide significant loci associated with ΔNIHSS24 h (Figure 1) [23]. These results explained only 1.8% of the variance in ΔNIHSS24 h. However, they also found that excitotoxicity could play an important role in neurological deterioration after stroke. Two genes that encode for synaptic proteins, namely *ADAM23* and *GRIA1*, which are enriched in neurons, were found to be associated with ΔNIHSS24 h and excitotoxicity.

Two GWAS have examined long-term outcomes after ischaemic stroke using the mRS at 3 months after stroke onset. In the GODS (Genetic contribution to Functional Outcome and Disability Study) study, the authors examined 2482 individuals and found that a single nucleotide variant in the *PATJ* gene was associated with long-term outcome (Figure 1) [22]. InaD-like protein (Inadl), encoded by *PATJ*, is localized to tight junctions in the apical membrane of epithelial cells [25]. The authors suggested that Inadl could be associated with blood–brain barrier homeostasis [22] and that regulation of the barrier could be the link between *PATJ* and stroke outcome.

The second GWAS included 6165 patients from the GISCOME (Genetics of Ischaemic Stroke Functional Outcome) network [21]. The phenotype used in this study was a dichotomized modified Rankin Scale score measured between 60 and 190 days after stroke onset. The authors found a significant genome-wide polymorphism at *LOC105372028* (Figure 1). This locus is a quantitative trans expression trait locus for *PPP1R21*, which encodes a regulatory subunit of protein phosphatase 1, and has been implicated in brain plasticity.

Although GWAS has the potential to uncover genetic mechanisms involved in stroke evolution and recovery, further GWASs are still required to better understand acute and long-term evolution. For future studies, it will be essential to standardize and correctly design the definition of phenotypes and endophenotypes and to take into consideration the parameters to be measured. In this regard, the Global Alliance of Acute and Long-Term Outcome genetics is an initiative from the ISGC to establish recommendations for genetic studies in stroke outcome. This initiative has published a guide with recommendations for genetic studies in stroke outcome [26].

## 4. Multi-Trait Analysis of GWAS

The standard approach in genome-wide association studies is to analyze one trait at a time. Although this is the ideal strategy, it is highly dependent on sample size and is not informative as to the possible pleiotropic loci with related phenotypes. A solution to this is to perform multi- or cross-trait analysis. This approach will allow us to increase the sample size by using genetically correlated traits, thereby enhancing the statistical power to detect new signals. This strategy is particularly interesting in the context of a complex disease such as stroke, where multiple intermediate phenotypes may be playing an important role.

In recent years, multi-trait analysis in GWASs has evolved to improve the performance of these methods. One major improvement was the use of summary statistics, which has significantly enhanced the power of multi-trait analyses given the availability of thousands of summary statistics in different repositories. Another major advance in the field was the use of traits with known or unknown sample overlap, which can be the case for multiple studies in different biobanks [27].

Different studies have explored the relationship between stroke and related phenotypes. Examples include the joint analysis of stroke and chronic obstructive pulmonary disease and stroke and major depressive disorder [28,29], thereby expanding our understanding of the shared genetic components of these traits.

Multi-trait analysis in GWASs has been most extensively studied in the lacunar stroke subtype as a phenotypic relationship is known to exist between this subtype and the presence of white matter hyperintensities in the MRI. A multi-trait analysis revealed seven additional loci to those found only in the single-trait study. The prioritized genes and associated loci were *SLC25A44-PMF1-BGLAP*, *LOX-ZNF474-LOC100505841*, *FOXF2-FOXQ1*, *VTA1-GPR126*, *SH3PXD2A*, *HTRA1-ARMS2*, and *COL4A2*. Interestingly, two of the loci identified contain genes (*COL4A2* and *HTRA1*) that are implicated in monogenic lacunar stroke, thereby highlighting the power of this multi-trait approach [30]. Another study also used lacunar stroke risk information, but in this case to potentiate genomic loci associated with intracerebral hemorrhage, as these two phenotypes are two diverse manifestations of small vessel disease. In this case, two new loci associated with non-lobar intracerebral hemorrhage were found, and a previous locus was confirmed [31].

A recent study has further explored the genetic relationship between CES and atrial fibrillation, the most prevalent risk factor for this subtype [32]. In this study, joint analysis with atrial fibrillation allowed the identification and replication in an independent cohort of eight novel loci associated with cardioembolic stroke risk, namely *CAV1*, *IGF1R*, *KIAA1755*, *NEURL1*, *PRRX1*, *SYNE2*, *TEX41*, and *WIPF1*. Additionally, it revealed loci associated with atrial fibrillation that do not increase the risk of CES, thus highlighting biological pathways with potential impact for the diagnosis of CES [33].

Finally, the GIGASTROKE study also explored the MTAG approach by adding the genetic information for related traits such as coronary artery disease, atrial fibrillation, and white matter hyperintensity volume. These analyses led to the identification of 24 novel loci associated with stroke and its subtypes [15].

All these published studies highlight the potential of the multi-trait approach to better understand the genomic architecture of stroke and to find new stroke-associated loci and related phenotypes.

## 5. Mendelian Randomization in Stroke

Inferring causality from observational studies is challenging due to the high possibility of bias. Randomized controlled trials are the gold-standard study design for determining the causal status of risk factors. However, as this approach has some limitations (i.e., time-consuming, expensive, and potentially unethical), alternative approaches are required. Mendelian Randomization (MR) is a statistical method that uses genetic variants to determine and quantify causal relationships between the effect of exposure on disease outcome [34].

MR studies use genetic variants to form subgroups analogous to those in a randomized control trial, although in this scenario the subgroups differ only in terms of exposure and not in any other factor, except for those that are causally linked with that exposure. Given the correct MR assumptions, a given genetic polymorphism that is strongly associated with a risk factor can be used to estimate the relationship between the risk factor and an outcome. An association between the genetic polymorphism and the outcome will only be possible if risk factor is causally associated with the outcome.

The increase in the availability of data from genetic studies has facilitated an upsurge in MR studies. In recent years, several MR studies have considered the previously described associations between cardiovascular risk factors and stroke in a search for causal effects [35,36]. An in-depth list of MR studies of IS can be found in Table 1 and Table 2.

Thus far, the take-home message from these studies is that the most well-established risk factors associated with stroke risk (atrial fibrillation, systolic and diastolic blood pressure, type 2 diabetes, smoking, obesity, etc.) do indeed play a causal role in IS risk and most of its subtypes [37,40,45,48,49,50,51,54,56,58,59]. Few studies have led to inconclusive results, as in the case of high-density cholesterol (HDL-C) [41,42,45].

As far as other diseases are concerned, there is no evidence of a causal association between depression and risk of IS [100,101], whereas one study has observed an association with increased risk of small vessel stroke (SVS) [100]. No causal associations were identified between migraine and AIS or stroke subtypes [102]. Impaired renal function is causally associated with an increased risk of LAS, but not other subtypes [110]. The circulating thyrotropin level has been causally associated with a lower risk of IS [76], while serum testosterone levels are a causal risk of IS [77].

With regard to lifestyle factors, such as physical activity, sedentary lifestyle, sleeping habits [57,61,62,63,64,65], and dietary habits [70,71,73], there is a general lack of evidence about their causal effect on IS. The exceptions to this are alcohol consumption [57,66,67] and insomnia [65], which are causally associated with IS and LAS, respectively, whereas following a Mediterranean diet appears to be protective against IS [106], and education level [57,103,104,105] and tea consumption are causally associated with a lower risk of SVS [68]. Anthropometric measures have also been studied, with results depending on the trait concerned. For instance, taller people have a lower risk of IS [108], whereas childhood obesity is causally associated with IS, LAS, and SVS [107].

An interesting approach is to study the effect that the microbiome has on the risk of IS. As the field develops, more MR studies will appear. To date, there is only one study, which did not find any evidence supporting a bidirectional causal relationship between gut microbiota-related metabolites and the risk of IS [99].

Perhaps the most interesting studies are those assessing causality using blood biomolecules as these are easy biomarkers to capture for stroke risk assessment but can also be potential drug targets. Not all the studies to date have observed causal associations with stroke risk, in either direction (inflammatory biomarkers [76,77,79,83], between circulating cytokines [96], vitamins [78,79,80,81,82,83,114], and many polyunsaturated fatty acids [70]). Some interesting findings are: (1) the identification of a causal link between lower serum MMP-12 levels and the risk of AIS, lower serum MMP-1 and MMP-12 levels and the risk of LAS, and higher serum MMP-8 levels and the risk of SVS [94]; (2) genetically determined levels of hemostatic factors have also been associated with the risk of IS [87,88]; (3) iron factors are causally associated with an increased risk of IS and CES, except transferrin, which is protective against IS and CES [73]; (4) among the cytokines studied, monocyte chemoattractant protein-1 (MCP-1) is the only one that was associated with an increased risk of IS, LAS, and CES [96].

### Conclusion

Many MR studies have observed estimated causal effects of a wide range of exposures on the risk of ischaemic stroke. MR studies are a powerful tool to confirm or refute the associations observed by GWAS, thus providing an extra tool for identifying the biological mechanism of stroke risk as well as novel therapeutic targets.

## 6. Stroke Pharmacogenomics

Different treatments are administrated in IS patients depending on the stroke stage and etiological stroke subtype. Currently, in the acute phase, only thrombolytic drugs, mostly recombinant tissue Plasminogen Activator (rtPA), are administrated [115]. Once the acute phase has passed, the secondary treatment is primordial [115] given that patients with a first stroke have twice the risk of suffering a new recurrent stroke [116]. This treatment depends on the etiological stroke subtype. Atherothrombotic IS is usually treated with antiplatelet drugs combined with lipid lowering drugs (mostly statins), while CES patients are usually treated with oral anticoagulants [115,116]. Although all these treatments are effective and safe, they are subject to inter-individual variation. Among the different variables contributing to this variability, genetics is a key factor affecting the drug response in IS patients. Since 2007, many GWASs analyzing the inter-individual variability in drug response have been published [117]. Compared with previous studies based on gene candidates, GWASs have allowed the detection of novel and unexpected genes with large effect sizes associated with drug response [117]. Drug selection based on the trial-and-error method is a costly approach that has led to efficacy and safety drug failures. The use of personalized medicine, based on individual genetic information, can help to select the appropriate drug for each patient, thereby improving the efficacy, safety, and cost of treatments [118,119].

In this section, different pharmacogenomic studies based on GWAS for the most widely used drugs in stroke will be reviewed.

### 6.1. Acute IS

rtPA is a thrombolytic drug used for clot lysis that is widely prescribed in IS patients who comply with specific criteria. However, although rtPA is effective, it is associated with an increased risk of hemorrhagic transformation (HT) and a lack of early recanalization in some patients [120]. Parenchymal hematoma (PH) is the most severe subtype of HT and is associated with higher death and disability rates. In a GWAS study of PH cases treated with thrombolytic recombinant tissue-plasminogen activator (rtPA) (*n* = 1324), the authors identified a significant genome-wide variant (rs76484331) located in the *ZBTB46* gene (Figure 1) [121]. *ZBTB46* encodes for a transcription factor expressed in the brain. The authors also observed how a polygenic risk score with 3506 variants from PH analysis was associated with disability and mortality at 3 months.

In another meta-analysis published involving 216 PH cases and 1818 controls, the gene *RP11-362K2.2: RP11-767I20.1* (Figure 1) was associated with the occurrence of PH. In addition, genetic correlations also found a shared genetic background of PH with Alzheimer’s disease and white matter hyperintensities [122].

### 6.2. Secondary Prevention: Oral Anticoagulants

Oral anticoagulants (OA), classified into vitamin-K antagonists (VKAs) and direct oral anticoagulants (DOACs), are widely prescribed for primary and secondary prevention of IS in patients with non-valvular atrial fibrillation (NV-AF) [122,123]. In Caucasian patients, there is up to a 20-fold inter-individual variation in the VKA dose required [124], therefore the international normalized ratio (INR) needs to be monitored continuously to assess the anticoagulation effect achieved by VKAs [125]. Low INR values indicate excessive anticoagulation, which leads to hemorrhage, and high INR values indicate insufficient coagulation, which is associated with loss of efficacy [124]. In DOACs, the mean variability values for peak and trough DOAC concentrations were calculated to be 34% and 36.6%, respectively. These inter-individual variations were not explained by renal clearance, thus other factors, such as genetics, are likely to have an important effect [126]. Different GWAS have been conducted in VKAs, most of them with warfarin and one with acenocoumarol. Only one GWAS has been published for DOACs (with dabigatran).

#### 6.2.1. Warfarin

A GWAS in Caucasian patients confirmed previous associations with the warfarin maintenance dose from candidate gene studies in *VKORC1* and *CYP2C9* [124,127]. The vitamin K epoxide reductase complex encoded by *VKORC1*, is involved in recycling from the inactive to the active form of vitamin K. Vitamin K is inhibited by warfarin and is a cofactor in the activation of coagulation factors [14]. *CYP2C9*, which encodes for a member of the cytochrome P450 family, is responsible for the metabolism of coumarins [128]. The variation in warfarin dose explained by *VKORC1* and two *CYP2C9* polymorphisms (CYP2C9*2 and CYP2C9*3) was 30% and 12%, respectively [10]. *CYP2F2* was also associated with warfarin dose after adjustment for *VKORC1* and *CYP2C9* variants [124].

The implication of *VKORC1* and *CYP2C9* in warfarin dose requirements was confirmed in other populations, namely African Americans [129,130], Brazilians [131], Japanese [132], and Middle East and North Africans [133]. Apart from these known associations, novel polymorphisms were found in African Americans—rs12772169, single nucleotide polymorphisms (SNPs) in *COX15* and *FGF5* [129], and an additional cluster in *CYP2C* in chromosome 10—associated with a decrease in the mean weekly dose [130]. In Japanese patients, *CYP2F2* was also associated with inter-individual warfarin variability [132].

The effect of genetics on bleeding events in patients treated with this drug was also assessed. Four genetic variants increasing the expression of *EPHA7* gene were found in the African American population, where the incidence of bleeding in patients treated with warfarin is higher [134].

#### 6.2.2. Acenocoumarol

Teichert et al. performed a GWAS in Caucasian patients from the Netherlands treated with acenocoumarol [135]. Polymorphisms in *VKORC1* and *CYP2C9* were associated with the acenocoumarol maintenance dose. Associations with *CYP2F2* and *CYP2C18* were also identified after adjustment for *VKORC1* and *CYP2C9* variants [135]. The association of polymorphisms in *VKORC1* and *CYP2C9* with acenocoumarol was also tested in a Spanish cohort of stroke patients treated with acenocoumarol using GWAS data [136]. Both genes were found to be associated with maintenance dose. Interestingly, some polymorphisms in these genes were associated with stroke recurrence and intracerebral hemorrhage.

The results from a coumarin GWAS resulted in a change in its labelling to indicate the recommendation for genotyping before starting treatment [128].

#### 6.2.3. Dabigatran

Paré et al. investigated whether some genetic component accounted for the concentration of the active dabigatran metabolite and whether these genes were also implicated in the efficacy and safety of the drug using GWAS [137]. Polymorphisms in *CES1* were associated with trough and peak metabolite concentration, while *ABCB1* was found to be associated with peak concentration only. *ABCB1* is important for the entry of dabigatran-etexilate (prodrug form) into the blood and *CES1* metabolizes dabigatran-etexilate to dabigatran to activate the drug [137]. In this GWAS, the *CES1* polymorphism, which is associated with a 15% reduction in the trough metabolite concentration, was also linked with a decrease in any bleeding risk. Subsequently, an in vitro study in liver samples demonstrated that *CES1* polymorphisms, coupled with sex, are associated with the variability in dabigatran activation [138].

### 6.3. Secondary Prevention: Antiplatelet Drugs

Antiplatelet drugs are administrated for the primary and secondary prevention of IS. The most widely prescribed such drugs are acetyl salicylic acid and clopidogrel. Acetyl salicylic acid has been the gold standard antiplatelet drug [139] since its efficacy was demonstrated for the first time in a clinical trial in 1978 [116]. Subsequently, in 1996, clopidogrel was also found to be effective [116].

#### 6.3.1. Acetyl Salicylic Acid

Acetyl salicylic acid inhibits platelet activity and aggregation by inhibiting the acetylation of cyclooxygenase-1 (COX-1) [118,139]. However, about 5–45% of patients treated with acetyl salicylic acid are resistant to the treatment and they do not achieve appropriate antiplatelet effects with it [139].

In a GWAS of acetyl salicylic acid response, the 1q23 locus (in *PEAR1* gene) was found by Lewis et al. to be associated with platelet response after dual antiplatelet treatment with acetyl salicylic acid and clopidogrel. Carriers of the A allele in the *PEAR1* polymorphism have an increased risk of suffering a cardiovascular event and death [140]. Stimpfle et al. found similar results for *PEAR1*, with this gene being associated with cardiovascular outcomes in high-risk patients undergoing a percutaneous coronary intervention [141]. In another GWAS, one polymorphism near *BCHE* was identified to be associated with plasmatic acetyl salicylic acid hydrolysis, which is important for acetyl salicylic acid inactivation [142].

#### 6.3.2. Clopidogrel

Clopidogrel is administrated as a prodrug that targets the P2Y12 receptor on the surface of platelets [118]. A third of the patients treated did not have enough platelet reactivity after a normal loading dose of 300 mg [143].

Different GWAS have investigated genetic associations with clopidogrel response in the Caucasian population. The 10q24 locus, including the cluster of *CYP2C18*, *CYP2C19*, *CYP2C9,* and *CYP2C8* genes, was associated with low clopidogrel doses in the first clopidogrel GWAS [144]. Other GWASs have linked *CYP2C19* variants with response to clopidogrel and with plasma levels of the drug [144,145,146,147]. The stronger variants associated with low response to clopidogrel and with platelet aggregation variation are CYP2C19*2 and CYP2C19*3 [118,144]. CYP2C19*2 has been found to be associated with a higher probability of cardiovascular ischaemic event or death [144]. Two additional loci, in 3p25 and 17q11, were associated with circulating clopidogrel levels as well as with platelet aggregation [145].

The International Clopidogrel Pharmacogenomics Consortium was created with the aim of determining the genetic basis for the inter-individual variability of the clopidogrel response [147]. This consortium included 17 studies from 13 countries to form the largest sample size achieved in a pharmacogenetic study of clopidogrel (8829 participants). Their GWAS only identified the signal in CYP2C19*2 associated with platelet reactivity [146].

Given the different implications of polymorphisms in different populations, one GWAS reported genetic associations with the effect of antiplatelet treatment with clopidogrel in a Chinese population with coronary heart disease [148]. *SLC14A2*, *ABCA1,* and *NGAMT1* were associated with the *P2Y12* reaction unit and with the active metabolite plasma concentration. The genetic variants in *NGAMT1* were found to be associated with major adverse cardiac events [148].

To overcome the issues caused by *CYP2C19* associated with lower activity of clopidogrel, the U.S. Food and Drug Administration added a warning to the summary of product characteristics regarding the relevance of considering the *CYP2C19* genotype in patients treated with clopidogrel [118].

### 6.4. Secondary Prevention: Statins

Statins are also widely prescribed in stroke patients. They are used for primary and secondary prevention due to their effect in the lowering of low-density lipoprotein cholesterol (LDL-c) [149,150,151,152]. They also present high inter-individual differences in terms of how they reduce LDL-c levels [153]. Different GWASs have identified some loci associated with different statins. The most strongly associated genes from different GWASs are *APOC1*/*APOE* [154,155,156,157], *LPA* [155,156,157], *SORT1*/*CELSR2*/*PSRC1* [155], and *SLCO1B1* [155,157].

## 7. Future Omics Studies in Stroke Research

The future in stroke research will depend on the ability to combine information from multiple omics technologies to obtain a complete map of the biological mechanisms associated with stroke risk and outcome. Several of these omics, such as metabolomics, lipidomics or single cell transcriptomics, are taking their first steps in stroke research. We will describe two of these fields that we think will provide interesting results in the stroke field in the near future.

### 7.1. Stroke Epigenetics

Epigenetics is the study of changes in gene function via regulation of gene expression. Epigenetic changes are heritable and reversible but do not involve changes to the DNA sequence. Epigenetics includes several regulatory systems, most prominently DNA methylation, histone modifications, and noncoding RNA.

In contrast to the genome, the epigenome varies throughout a person’s life and from cell-type to cell-type. Indeed, epigenetic modifications play an important role in the pathogenesis of many complex diseases [158], including stroke [159].

#### 7.1.1. DNA Methylation

DNA methylation (DNAm) involves the addition of a methyl group to the cytosines of cytosine-phosphate-guanine (CpG)-rich segments in DNA and methylation profiles in the promoter region or gene body have different effects on expression. Several studies have revealed altered methylation profiles in IS patients compared to controls in terms of global methylation proxies [160,161,162] or epigenome-wide association studies (EWAS) [163,164]. In a stroke-obesity cohort (*n* = 139 patients), methylation changes in 80 cytosine-guanine dinucleotides (CpG) were observed (*p*-value = 0.05). Further analysis identified obesity-induced changes in the *KCNQ1* methylation level related to stroke risk [163]. A subsequent two-stage study (*n* = 793) identified 384 differentially methylated CpGs, with 22 of these being further validated [164]. The location of these CpGs suggested a possible implication with gene expression, and nearby regions previously associated with IS in genetic studies [164].

Evidence for specific DNAm signatures for different stroke subtypes is still weak. One study using the luminometric methylation assay as a proxy for global methylation, did not identify methylation differences between IS subtypes [165]. A more recent study, using an EWAS approach, identified changes in *MTRNR2L8* methylation as a potential biomarker for LAS stroke [166].

Specific DNAm profiles have been found to be predictors of aging, generating predictor DNAm age, a proxy for chronological age [167,168]. The DNAm age of IS patients is higher than their chronological age and that for matched controls [169]. DNAm age is also a better predictor of IS mortality [170]. More recently DNAm age differences between men and women, at the time of stroke onset, have been reported [170].

#### 7.1.2. Histone Modifications

Histone proteins are the packing blocks for DNA. Histone modifications may regulate access to transcriptional factors, and thus alter gene expression, by opening or tightening the chromatin [171]. The most commonly studied histone modification is acetylation/deacetylation of the lysine residue of histones H3 and H4. Histone acetyltransferases (HATs) are responsible for acetylation, while histone deacetylases (HDACs) catalyze the deacetylation.

There is a general increase in acetylation of H3 and H4 in animal models of stroke [172,173]. Thus, HDAC inhibitors have been thoroughly studied in in vitro and in vivo animal models to investigate their potential role in preserving or restoring the normal acetylation levels of H3 and H4 [174,175,176,177,178,179]. However, the beneficial effect in patients is lacking, as there are no phase III clinical trials assessing the viability of HDAC inhibitors in managing stroke [180]. Interestingly, an intronic variant in HDAC9 has been associated with the risk of LAS in several studies [2,15,181].

#### 7.1.3. Non-Coding RNA

Non-coding RNA (ncRNA) is RNA from non-coding regions of the genome. In recent years, it has been proposed to play a role as a regulatory element involved in DNA methylation, acetylation, alternative splicing, and post-transcriptional modifications, amongst others. Micro RNAs (miRNAs) are the best characterized ncRNA subtype.

Various miRNAs have been associated with IS, thus suggesting a potential diagnostic value (miRNA-335 [182], miRNA-424 [183], miRNA-15a [184], and miRNA-429 [185] amongst others). In a study of 745 miRNAs from serum samples, 71 miRNAs were up-regulated and 48 down-regulated, of which four (miR-23b-3p, miR-29b-3p, miR-181a-5p and miR-21–5p) were validated to be elevated in IS. Based on serum miR-23b-3p, miR-29b-3p, and miR-21–5p levels, the authors were able to discriminate between IS and TIA patients [186].

The reversible nature of epigenetic modifications makes them an important target for the development of new treatments. Epigenetic therapies have been largely studied in cancer research, leading to licensed anticancer epigenetic treatments. Similar approaches are also explored for cardiovascular diseases, such as atherosclerosis [187], myocardial infarction [188], and atrial fibrillation, [189] (ClinicalTrials.gov Identifier: NCT03298321) including ischaemic stroke [190].

Epigenetic studies are currently mostly performed in small sample sizes and methodologies are inconsistent. This challenges the ability to replicate the findings. Further efforts in this field are therefore required to validate the results obtained.

### 7.2. Gut Microbiome Studies

The estimated total cell count of a typical human body is 6.8 × 10^13^. However, 3.8 × 10^13^ of these cells are bacteria that live within us [191], thus meaning that we are composed of only 44% human cells. At the same time, the genome size of the human gut microbiome is 150 times larger than that of the human genome [192]. All this genetic content from the microorganisms that inhabit our body is called the metagenome.

Recent advances in next-generation sequencing technology have allowed researchers to study metagenomics and to investigate all the genetic material comprising the human-associated microbiota in greater depth. This has led researchers to gain a better understanding of the gut–brain axis and its implications for modulating host inflammation in some diseases such as type 2 diabetes [193], obesity [194], and atherosclerosis [195], among others.

With regard to IS, multiple studies have shown a significant gut dysbiosis and lower trimethylamine-N-oxide (TMAO) levels in stroke and transient ischaemic attack (TIA) patients compared with a healthy population [196,197]. Despite this, it is known that there is a robust association between increased levels of this metabolite and future risk of stroke [198] by promoting atherosclerosis and being associated with platelet hyper-reactivity and inflammation [199]. Therefore, these new results suggest that either the stroke event or its treatment may reduce TMAO [196], and further research about the potential therapeutic strategy in reducing TMAO levels is therefore needed.

Furthermore, this gut dysbiosis is characterized by a loss of alpha-diversity in stroke patients [200,201], although one study found the opposite [196], and significant differences in alpha-diversity between the IS group and controls [202].

The gut microbiota in IS patients was also distinguished by an enrichment of opportunistic pathogens from genera such as Enterobacter, Megasphaera, and Desulfovibrio, and a depletion of beneficial microbes such as Bacteroides, Prevotella, and Faecalibacterium [196]. Additional research has shown that Enterobacteriaceae family overgrowth may play a role by accelerating systemic inflammation after stroke [197].

In addition, differences in the severity of IS, as classified using the NIHSS score, have also been associated with gut dysbiosis. These differences include depleted levels of Bacteroides in severe stroke (NIHSS score > 4) patients compared with mild stroke (NIHSS score ≤ 4) patients [196], higher abundance of Escherichia/Shigella in severe stroke patients [196], and an increase in Enterobacter, Pyramidobacter, and Lachnospiraceae UCG-001 in mild stroke patients [203].

Furthermore, the evolution of short- and long-term functional outcomes after an IS has been associated with the relative abundance of the genus Roseburia, thus suggesting a protective role for this bacterium in stroke evolution and outcome [204]. A long-term poor functional outcome has been associated with reduced short-chain fatty acid-producing (SCFA) bacteria [205].

More recently, metagenomic studies have been combined with genome-wide association studies (GWAS). This has allowed the relationship between the gut microbiome composition and host genetics to be identified. Kurilshikov et al. analyzed 18,340 individuals from cohorts with multiple ancestries and found an association between Bifidobacterium and the LCT locus [205].

Another GWAS performed in 8956 German individuals identified significant associations between Bacteroides and Faecalibacterium spp. and the *ABO* gene, and a possible association between Bacteroides and the FUT2 locus [206].

In Table 3 appears the studies assessing the microbiota-gut-brain axis in IS.

Ultimately, all these findings may allow improvements in bacteriotherapy, and some of them have already been tested in animal models. Lee et al. found that poor stroke recovery in aged mice could be reversed with post-stroke fecal transplants from youthful microbiota [207], thus proving that further research into the human microbiome might benefit targeted therapy and personalized medicine.

## 8. Conclusions

The combination of GWAS data with bioinformatic analysis is a powerful tool to understand the biological mechanisms of complex diseases. Moreover, the initiatives to make genetic and other “omic” data available for public research is resulting in an opportunity to improve and accelerate research in common and rare diseases, especially those with a heritability component.

Several bioinformatic tools using genetic data can give useful information for research in other specialties, including clinical research. For example, Mendelian Randomization is an interesting tool to estimate causality among diseases and traits. Moreover, genetic data can be useful for finding potential drug targets and potential treatments using currently available drugs.

Future studies using genetics and other omics will focus on acute and long-term stroke outcome to find potential drugs to prevent stroke disability and mortality. Maybe where previous studies over the past decade have failed, genetics could be the key to finding genuinely useful treatments to improve stroke outcome.

It should be emphasized that other omics, such as epigenetic studies or microbiome analysis, do not have the same trajectory as genetic studies, with most of the studies analyzing small sample sizes and, consequently, providing inconsistent results. However, these omics, combined with genetic and clinical data, will be very important in understanding the mechanisms of stroke. Moreover, the reversible nature of epigenetics, transcriptomics or microbiomics makes this an attractive field of research as they can potentially be modulated to improve stroke risk and outcome.

## Figures and Tables

**Table 1:** Results from MR studies involving traditional stroke risk factors.

Exposure			AIS	LAS	CES	SVS	References	
SBP							[37,38]	
DBP							[37,38]	
Mean Arterial Pressure	(≤55 y)						[39]	
	(>55 y)						[39]	
Pulse Pressure	(≤55 y)						[39]	
	(>55 y)						[39]	
Atrial Fibrillation							[37]	
				-	-	-	[40]	
LDL-c							[41]	
				-	-	-	[42]	
							[43]	
		EAS		-	-	-	[44]	
		AFR		-	-	-	[45]	
HDL-c							[41]	
				-	-	-	[42]	
		AFR		-	-	-	[45]	
Lp(a)							[46,47]	
T2DM							[48]	
							[49]	
				-	-		[50]	
		EAS					[51]	
		AFR		-	-	-	[45]	
HbA1c							[49,52]	
Insulin resistance							[49,53]	
Fasting insulin							[48]	
β-Cell dysfunction							[49]	
Smoking							[54]	
Smoking initiation							[55,56]	
Lifetime smoking							[56,57]	
General adiposity (BMI)							[48,57,58,59]	
Central adiposity (WHR)							[57,58,59]	
Childhood obesity							[60]	
Physical activity							[57,61,62]	
Sedentary behavior				-	-	-	[61,62]	
High risk (HR)			Low risk (LR)				no evidence	
Inconsistent HR assoc.							Not studied	-

AIS, all ischaemic stroke; LAS, large artery stroke; CES, cardioembolic stroke; SVS, small vessel stroke; EAS, East Asian population; AFR, African population; SBP, systolic blood pressure; DBP, diastolic blood pressure; HDL-c, high-density lipoprotein cholesterol; LDL-c, low-density lipoprotein cholesterol; Lp(a), lipoprotein (a); T2DM, type 2 diabetes mellitus; HbA1c, Glycated hemoglobin; BMI, body mass index; WHR, waist-to-hip ratio.

**Table 2:** Results from MR studies involving non-traditional stroke risk factors.

Exposure				AIS	LAS	CES	SVS	References	
Sleep Duration								[57,61,63]	
Sleep Duration	short (<7 h)							[63,64,65]	
Sleep Duration	Long (≥9 h)							[63,64,65]	
Continuous sleep								[65]	
Chronotype (morningness)								[65]	
Insomnia symptoms								[65]	
Alcohol								[57,66,67]	
Tea								[68]	
Coffee								[57,69]	
PUFA	Linoleic acid (LA)				-			[70]	
	Arachidonic acid (AA)				-	-	-		
	α-linolenic acid (ALA)				-	-	-		
	Eicosapentaenoic acid (EPA)				-	-	-		
	Docosahexaenoic acid (DHA)				-	-	-		
	Docosapentaenoic acid (DPA)				-	-	-		
Urine sodium								[71]	
Serum magnesium								[72]	
Serum calcium								[72]	
Iron status	Iron							[73]	
	Ferritin								
	Transferrin saturation								
	Transferrin								
Serum bilirubin levels		EAS			-	-	-	[74]	
Uric acid								[75]	
Thyroid hormones	Thyrotropin (TSH)				-	-	-	[76]	
	Free thyroxine (FT_4_)				-	-	-		
Serum testosterone					-	-	-	[77]	
Vitamin D (25OHD)					-	-	-	[78,79]	
Vitamin K_1_								[80]	
Vitamin C								[81,82,83]	
Homocysteine								[84,85,86]	
Hemostasis traits	FVIII activity							[87]	
	FVIII antigen								
	FXI activity								
	FXI							[88]	
	Gamma fibrinogen							[87]	
	TAFI-AP antigen								
Hematological traits	Plateletcrit							[87]	
	platelet count							[87,89]	
	Eosinophil percentage							[87]	
Inflammatory		CRP						[90,91,92]	
Biomarkers		TIM-1			-	-	-	[93]	
		sIL-6R						[91]	
Matrix Metalloproteinases		MMP-1						[94]	
		MMP-8							
		MMP-12							
Circulating cytokines		IL-1ra						[91,95]	
and growth factors		IL-2ra						[95,96]	
		IL-5						[96]	
		IL-6						[95]	
		IL-10						[96]	
		IL-12p70						[96]	
		IL-16						[95,96]	
		IL-17						[95,96]	
		IL-18						[95,96]	
		CTACK						[96]	
		BNGF							
		Eotaxin							
		GDF-15						[97]	
		GRO-α						[96]	
		HGF							
		IP-10							
		MCP-1							
		MIF							
		MIG							
		MIP-1b							
		PDGF-bb							
		SCF							
		SCGF-b							
		TNF						[98]	
		TNF-β						[96]	
		TRAIL							
		VEGF							
Genetically downregulated IL-6 signaling								[92]	
NO signaling					-	-	-	[8]	
Gut microbiota dependent metabolites					-	-	-	[99]	
Major depressive disorder								[100]	
Depression					-	-	-	[101]	
Migraine								[102]	
Education								[57,103,104,105]	
Metabolic signature of Mediterranean diet					-	-	-	[106]	
Lower birth weight								[107]	
Height					-	-	-	[108]	
Resting heart rate								[109]	
Impaired renal function								[110]	
Periodontitis								[111]	
Telomere length			EAS					[112,113]	
High risk (HR)				Low risk (LR)				no evidence	
Inconsistent HR assoc.				Inconsistent LR assoc.				Not studied	-

AIS, all ischaemic stroke; LAS, large artery stroke; CES, cardioembolic stroke; SVS, small vessel stroke; EAS, East Asian population; 25OHD, 25-hydroxyvitamin D; BNGF, beta nerve growth factor; CRP, C reactive protein; CTACK, cutaneous T-cell-attracting chemokine; FXI, coagulation factor XI; GDF-15, growth differentiation factor-15; GRO-α, growth-regulated oncogene alpha; HGF, hepatocyte growth factor; IL, interleukin; IL, interleukin; IL-1ra, interleukin 1 receptor antagonist; IL-2ra, interleukin 2 receptor antagonist; IP-10, interferon gamma-induced protein 10 MCP-1, monocyte chemoattractant protein-1; MIF, macrophage migration inhibitory factor; MIG, monokine induced by gamma interferon; MIP-1b, macrophage inflammatory protein 1 beta; PDGF-bb, platelet-derived growth factor-bb; PUFA, poly-unsaturated fatty acids; SCF, stem cell factor; SCGF-b, stem cell growth factor beta; sIL-6R, soluble interleukin 6 receptor; TAFI-AP, thrombin-activatable fibrinolysis inhibitor activation peptide; TIM-1, T-cell immunoglobulin and mucin domain 1; TNF, tumor necrosis factor; TRAIL, TNF-related apoptosis-inducing ligand; VEGF, vascular endothelial growth factor; NO, nitric oxide.

**Table 3:** Mentioned studies assessing the microbiota-gut-brain axis in ischaemic stroke.

	Study Design	Results
Xu et al., 2021 [196]	Gut microbiome studied in two human clinical cohorts. Mouse stroke model for ischaemic using a middle cerebral artery occlusion (MCAO).	Gut dysbiosis both in humans and mice after the ischaemic stroke. This dysbiosis is characterized by an overgrowth of Enterobacteriaceae.
Zhu et al., 2016 [197]	Association between plasma TMAO levels and incident thrombotic event risk in humans. Mouse stroke model using germ-free mice to confirm the role of TMAO modulating thrombosis.	Higher levels of TMAO predict incident risk for thrombotic events (myocardial infarction and stroke) in humans.
Singh et al., 2016 [198]	Gut microbiome studied in germ-free mice and mice models of MCAO. Fecal transplantation experiments.	Post-stroke dysbiosis is characterized by a reduced diversity and a Bacteroidetes overgrowth. Transplantation of fecal microbiota improves stroke outcome.
Haak et al., 2021 [201]	Prospective case-control study using ischaemic and hemorrhagic stroke patients and controls.	Disruption of gut microbiota during ischaemic and hemorrhagic stroke, characterized by an enrichment of bacteria implicated in TMAO production and a decrease of butyrate-producing bacteria.
Wang et al., 2018 [202]	Gut microbiome studied using fecal samples of healthy subjects and cerebral infarction (CI) patients.	CI patients have higher levels of Gammaproteobacteria and lower levels of Bacteroidia, which is correlated with ApoE levels in the serum.
Gu et al., 2021 [203]	Structure of fecal microbiome studied in acute ischaemic stroke (AIS) patients with minor and non-minor stroke.	Relative abundance of Roseburia is associated with severity of the AIS and short-term and long-term outcome.
Tan et al., 2021 [204]	Gut microbiome and SCFA studied in AIS patients and healthy controls.	AIS patients are characterized by a lack of SCFAs-producing bacteria. AIS patients have lower levels of SCFAs, which is negatively correlated with stroke severity and prognosis.
Lee et al., 2020 [207]	Fecal transplant using a mouse model for ischaemic stroke induced with MCAO.	Fecal transplant from young mice to aged MCAO mice can improve stroke recovery by modulating the immunologic, microbial, and metabolomic profiles in the host.
